# Potential Challenges of Controlling Leishmaniasis in Sri Lanka at a Disease Outbreak

**DOI:** 10.1155/2017/6931497

**Published:** 2017-05-28

**Authors:** Tharaka Wijerathna, Nayana Gunathilaka, Kithsiri Gunawardana, Wasana Rodrigo

**Affiliations:** ^1^Department of Parasitology, Faculty of Medicine, University of Kelaniya, Kelaniya, Sri Lanka; ^2^Biotechnology Unit, Industrial Technology Institute, Colombo 7, Sri Lanka

## Abstract

The present works reviewed the existing information on leishmaniasis in Sri Lanka and in other countries, focusing on challenges of controlling leishmaniasis in the country, in an outbreak. Evidence from recent studies suggests that there is a possibility of a leishmaniasis outbreak in Sri Lanka in the near future. Difficulty of early diagnosis due to lack of awareness and unavailability or inadequacy of sensitive tests are two of the main challenges for effective case management. Furthermore, the absence of a proper drug for treatment and lack of knowledge about vector biology, distribution, taxonomy and bionomics, and reservoir hosts make the problem serious. The evident potential for visceralization in the cutaneous variant of* L*.* donovani* in Sri Lanka may also complicate the issue. Lack of knowledge among local communities also reduces the effectiveness of vector and reservoir host control programs. Immediate actions need to be taken in order to increase scientific knowledge about the disease and a higher effectiveness of the patient management and control programs must be achieved through increased awareness about the disease among general public and active participation of local community in control activities.

## 1. Background

Leishmaniasis is a worldwide vector borne zoonotic disease caused by several heteroxenous intracellular parasitic organisms of genus* Leishmania*. The disease has several different forms including three main forms, namely, visceral leishmaniasis (VL), cutaneous leishmaniasis (CL), and mucocutaneous leishmaniasis (MCL), where VL is considered to be the most virulent form of the disease. Although several case reports provide evidences for sexual or vertical transmission [1–4], the main method of transmission of this disease is through the bite of the female sand flies of subfamily Phlebotominae.

Leishmaniasis has been listed as one of the eight major neglected tropical parasitic diseases with a prevalence in more than 90 countries where 88 of them are endemic to the disease. The zoonotic nature and the large genetic diversity of the both parasites and vectors make the control of this disease very difficult [5, 6].

In the control of leishmaniasis, early diagnosis and effective patient management, vector control, effective disease surveillance, control of reservoir hosts and social mobilization, and strengthening partnerships are the key strategies [6]. Early diagnosis and effective case management are essential to reduce the prevalence of the disease and prevent disabilities and death. There are no available vaccines or drugs to provide immunity to the infection. Therefore, the most effective way to prevent infection is prevention of sand fly bites by safety and vector control measures [5, 6].

## 2. Leishmaniasis in Sri Lanka and Challenges in Controlling

The first report of leishmaniasis from Sri Lanka was found in the 1990s. However, those were not due to local transmission, but reported mainly among overseas employees returning to the country [7]. First evidences for local transmission of leishmaniasis in Sri Lanka was in 1992 [8]. In 2008, this disease was named as a notifiable disease in Sri Lanka by the Epidemiology Unit of Sri Lanka [9]. Thereafter the disease was identified as a significant health problem in the country and a national action plan was adopted for the control of leishmaniasis [10]. All of the cases reported in past were cutaneous leishmaniasis. The first case of MCL was detected in 2005 [11] while the first patient with VL was identified 2007 [12]. At present, Sri Lanka is considered as a one of the endemic countries for CL [13] in the world and the newest reported focus of leishmaniasis in the Indian subcontinent where the disease is caused by the most virulent visceralizing species,* Leishmania donovani*. However, the potential for visceralization in the cutaneous variant of* L. donovani* in Sri Lanka is not known.

After 2001, an outbreak of CL in the island became evident, but still relatively an unknown disease [13]. However, VL and MCL have been reported less frequently than CL so far. The presence of vectors may increase the transmission risk and thereby the prevalence of the disease [14]. Studies have shown that the population of the vector species fluctuates with the seasonality (rainy or dry) and associated environmental factors [15]. In addition, the disturbances in environment which result in the loss of natural land coverage may also facilitate with favorable resting and breeding habitats for sand flies, thus increasing the probability of intensifying the sand fly population [14]. On the other hand, building constructions clearing original land coverage causes humans to get closer to living habitats of reservoir animals, further increasing the risk of infection. Increasing encroachment in to forest has resulted in such outbreaks in other countries [14, 16, 17].Therefore, this disease can be more serious if a sudden outbreak of vector population occurred.

When considering the progress of the disease during last sixteen years, with increasing encroachment in to forests and ever-changing environmental conditions due to climate changes, occurrence of a serious outbreak of leishmaniasis in Sri Lanka is very likely. Hence, this review article emphasizes the potential challenges of controlling leishmaniasis in Sri Lanka at a probable outbreak and the steps that need to be taken in order to minimize adverse impact.

## 3. Diagnosis of the Disease

Early diagnosis is a very important factor for an effective management of any disease. Currently, several parasitological, molecular biological, and serological tests are used for the diagnosis. However, the diagnosis of leishmaniasis is still controversial as none of the available tests provide 100% specificity and sensitivity. Also the symptoms may vary from one form to another and also can be similar to some other diseases [18]. The simplest diagnostic method of leishmaniasis is microscopic identification of amastigote form of the parasite on a stained smears of samples obtained from skin lesions, liver, spleen, lymph nodes, or bone marrow [10, 19].

Advanced costly techniques such as Polymerase Chain Reaction (PCR) are more sensitive than microscopic observations [19–22]. Immunological methods are also widely used for the detection of parasites in blood. Mainly, Indirect Fluorescent Antibody (IFA) test is used to detect anti-leishmanial antibodies. Enzyme Linked Immunosorbent Assay (ELISA) is another serological test which is more useful in detecting visceral leishmaniasis. Immunochromatographic tests such as rK39 test which is based on recombinant K39 antigen are also performed in the detection of leishmaniasis [13, 23]. In the diagnosis of CL, Leishmanin Skin Test (LST) can be used in order to measure delayed hypersensitivity caused by CL [24, 25]. Direct Agglutination Test (DAT) is relatively cheaper compared to other immunological tests mentioned above. However, this method is not appropriate since it has lower sensitivity [10, 19, 20, 22].

In Sri Lanka, the detection of the disease is passive and only the patients seeking medical attention for their symptoms are screened for the presence of parasites. No programs are conducted to detect patients at early stages of infection [13]. Detection mainly depends on microscopic observations of GIEMSA stained smears while advanced techniques such as PCR, ELISA, rK39, and other molecular diagnostic test are available in very limited laboratories [13, 26]. These limitations of facilities to perform more advanced and sensitive tests can become an indispensable problem in early diagnosis, patient management, and prevention of the transmission of the disease.

Microscopic detection of parasites in VL requires some techniques that are very difficult to perform. For an example spleen aspiration which has a relatively higher sensitivity may be very dangerous since it can cause fatal damage to spleen and thus requires well trained personnel to perform [19, 27, 28]. Other samples that can be obtained easily from bone marrow and peripheral blood have a low sensitivity [19, 27]. Although obtaining samples from skin lesions is much easier in CL, the results depend on the expertise of the observers and the quality of prepared slides [27]. Sensitivity can be improved by the culture of parasite, which in turn demands expensive facilities which is not available in a developing country like Sri Lanka and it is time consuming, thus reducing the effectiveness of the diagnosis [27, 29].

Other tests used in Sri Lanka, specially rK39 test, are inexpensive and rapid and can be performed by untrained person [19, 27, 30] but less reliable as the sensitivity has been reported to be dependent on the ethnic group [29]. Therefore, this test may pose a considerable challenge in effective patient management of leishmaniasis in Sri Lanka especially in an outbreak situation.

## 4. Treatments for the Disease

Immunization for the disease is not yet established since the unavailability of a vaccine, although it appears to be very effective in controlling the disease [31]. As in all other neglected diseases, leishmaniasis also has the problem of having a minimum attention by drug developers since the treatment essentially needs to be affordable to poor population. Therefore, development of a vaccine cannot be expected in the near future. Hence, the control of the disease is mainly based on systemic and local treatments using drugs and/or nonpharmaceutical methods.

Systemic treatments include the administration of drugs through oral, intravenous (IV), intramuscular (IM), or intralymphatic (IL) routes [32]. Administration of Pentavalent antimonial drugs such as Sodium stibogluconate through IV and IM routes is the main method of the treatments despite the clinical representation of the disease [5, 33–35]. Administration of amphotericin B or Liposomal amphotericin B through IV route is also done in leishmaniasis treatments [36–39]. However, use of Liposomal amphotericin B considered as a better option than the conventional amphotericin B [5]. Oral administration of miltefosine and the IM administration of paromomycin and pentamidine are also used for leishmaniasis [37, 40–42]. In addition, certain azole compound normally used as antifungal agents including fluconazole and itraconazole are also used in treatments for both CL and MCL [43, 44].

In local or topical treatments for CL, both drugs and nonpharmaceutical agents are used. The drug that is mostly used in topical treatments is paromomycin, while some other drugs such as imidazole and methylbenzethonium chloride [45–47]. Use of some drugs in combination with other drugs showed to be more effective than using them alone. For an instance El-On et al. [48] show that the use of paromomycin sulfate in combination with methylbenzethonium chloride may be more effective in the treatment for CL lesions. Another study has also shown that the use of these two drugs with Gentamicin is significantly more effective than using previous two alone [49]. Intralesion administration of drugs such as Sodium stibogluconate and meglumine antimoniate is also used in pharmaceutical local treatments [50, 51]. Certain studies suggest that the intralesional administration of Sodium stibogluconate is more effective than the most common cryotherapy and has less side effects [50].

When considering nonpharmaceutical topical treatments, cauterization or the application of heat to the site of the lesion is the simplest treatment. This can be very effective since* Leishmania* parasites are heat sensitive [52, 53]. However, cryotherapy using liquid nitrogen is the most commonly used method [54].

In Sri Lanka, intralesional administration of Sodium stibogluconate is used as the main method of treatment for CL, which is more common and known to have relatively less side effects [13]. However, practically it is not possible to use intralesional administration of drugs for all the situations. For an instance if the patient is a child and the lesion is at a sensitive part of the body, intralesional administration cannot be used. Then other methods such as heat treatments or cryotherapy should be used in based on the practical constraints. Another major problem is the high toxicity of drugs such as some antimonials used in treatments for all three forms of the disease. Any of the currently used antileishmanial drugs cannot be considered as the ideal drug for the disease due to high toxicity and the difficulty in administration [55, 56]. On the other hand the resistance to the drugs used for treatments of leishmaniasis is evident from some studies, mainly for some pentavalent antimonial drugs [40, 57].

Other disadvantages of currently available drugs are the requirement of long term treatments, variation of efficacy from region to region, and the possibility of the recurrence of the disease [32, 34]. On the other hand these treatments are not available in all the hospitals of the country. Patients must travel a long distance to those hospitals having dermatology clinics. Since most of these patients are farmers and daily workers, it creates considerable economic losses to their families. Therefore, the patient management will be a challenging task under these situations.

## 5. Presence of Vectors and Vector Control Approaches

Vector control is currently the most effective control strategy for leishmaniasis. Chemical, biological, and environmental methods are used to control leishmaniasis. When considering chemical control, indoor residual spraying is used worldwide. In Morocco, indoor residual spray has resulted in reducing disease incidence [58]. However, researches have shown that sand flies may develop resistance to these chemicals from both African and Asian countries [59, 60]. Therefore, main attention must be given toward environmental and biological control as well as prevention of bite of sand flies. Main method of environmental control is reducing the chance of breeding of sand flies altering environment to become unsuitable for breeding [61]. Normally sand flies breed in dark corners in the crevices of the walls having rich humus and moisture. Thus the most suitable way to control the disease is by preventing the breeding by plastering the walls [62] or by cement skirting on walls and the floor [63] which have experimentally proven to be effective in controlling sand fly populations. Certain biological control methods including the use of microorganisms to kill the larval stages are being tested and it has been proven to be effective, but the feasibility of practically use is yet to be evaluated [64–67].

However, controlling methods in many of the countries have been proposed and tested after thorough investigations on vector, for instance, exact vector species, behavior, breeding habits and places, resting places, distribution, and other bionomic characteristics such as population fluctuations. In Sri Lanka very few studies have been conducted on these aspects which is not sufficient to support for such control programs.

The most common vectors, Phlebotomine sand flies have a wide distribution in tropics and other warm mainland areas, and extend up to southern parts of the northern hemisphere [20]. There are more than 927 known species or subspecies of sand flies that have been identified so far around the world while only three genera,* Phlebotomus, Lutzomyia*, and* Sergentomyia, are* known to feed on vertebrate blood [20]. However, according to previous studies before 2014, only about 50 species of genera* Phlebotomus* and* Lutzomyia* are known to be responsible for the transmission of the disease to humans [68, 69], but complicating the issue even more, studies revealed that some species of genus* Sergentomyia* also have the ability to harbor* Leishmania* parasites suggesting a high likelihood of transmitting the disease by the species of the genus* Sergentomyia* [70, 71]. This is about to get worse as evidence is appearing to suggest that biting midges in the genus* Culicoides* also act as a host for the transmission of leishmaniasis [72]. However, the most common species of the genus,* P. argentipes*, is the proven vector of* L. donovani* which causes visceral leishmaniasis in the Indian subcontinent [73].

Presence of* P. argentipes*, the vector of* L. donovani* vector in other countries, has been reported from Sri Lanka in early 1940s [74]. Therefore, scientists suspected that* P. argentipes* is the vector of leishmaniasis in Sri Lanka. Interestingly, in 2009 studies revealed that all three members of* Phlebotomus* complex including,* P. glaucus, P. argentipes*, and* P. annandalei* are prevalent in Sri Lanka [75]. Molecular studies provide evidence for the presence of* Leishmania *DNA in blood fed and unfed female sand flies confirming the possibility of* P. argentipes* to act as vectors of leishmaniasis [76].

However, exact number of species that transmit the disease in Sri Lanka is yet poorly understood. Vector species range especially has been found to be broader than predicted earlier. For an instance, earlier only the genus* Phlebotomus* was known to act as vectors for* Leishmania* parasites, but as mentioned earlier, recent studies have proven the presence of* Leishmania* parasites in sand flies of the genus* Sergentomyia*. In Sri Lanka, 8 species from the genus* Sergentomyia* have been recorded [77–79]. Therefore, there is a possibility of these species acting as vectors in the country which is not yet discovered. Studies on biting midges and their potential capacity for the transmission of leishmaniasis also have rarely been studied in Sri Lanka. Hence, lack of knowledge on the species currently present from this group is a huge drawback as they apparently act as the vectors of leishmaniasis.

With the increasing complication of the issue, Sri Lanka has a huge challenge as the exact species that are transmitting the disease is yet unknown. On the other hand only a little knowledge is available on the species proven to be vectors in Sri Lanka, about their behavior, especially, breeding places, resting places, and the distribution which are essential in designing vector control strategies, and also there are no information on population dynamics of these sand flies which can be very useful in a sudden environmental change which may lead to an outbreak of sand flies and in turn of the disease, leishmaniasis.

In addition to control of sand fly populations, the prevention of sand fly bite is also important for effective control of the disease, for that also the information on biting times of sand flies and their habitat preferences which are very scanty for Sri Lankan species are needed.

## 6. Availability of the Reservoir Hosts

The infective agents of leishmaniasis are heteroxenous unicellular organisms where a single species of parasite can survive within several different hosts. Thus, the disease is a zoonosis with a range of sylvatic mammals, such as forest rodents, hyraxes, and wild canids and also domesticated animals mainly, dogs acting as reservoir hosts [80–85]. Reservoir hosts play an important role in the maintenance of the transmission cycle of the disease, because the cycle can be maintained by reservoir hosts, even in the absence of an infected human host [5]. Alterations in the range and density of the reservoirs, resulted from human made changes in the environment, may increase human exposure to infected sand flies [86]. Therefore, the zoonotic nature increases the prevalence of the disease.

Hence, the control and management of reservoir hosts is an effective control measure for leishmaniasis. For that the identification of exact species that act as reservoir hosts is essential. On the other hand their behavior, habitats, and specific habitat conditions are also needed to be understood in order to prevent the contact with humans. Screening of animal species individually for parasites is very difficult and time consuming. Hence, more effective methods such as screening blood fed sand flies for reservoir host DNA may give more reliable results in the identification of reservoir hosts [87, 88].

In Sri Lanka, few studies have been conducted to find out possible reservoir hosts for the disease and dogs have been identified as possible reservoir hosts [81, 89]. However, some rodent species have been screened for parasites but the results have been negative according to the study conducted by Nawaratna et al. [81]. When considering the huge diversity of sylvatic and peridomestic mammals that have been reported from other countries as reservoir hosts for the disease, any mammal can be a reservoir, but except some rodents, no wild animals have been screened in previous studies in Sri Lanka as it is very difficult due to locate and catch these animals, especially bats, forest dwelling cryptic rodents, and the only wild canid in Sri Lanka, “the jackal” which has a possibility of acting as a reservoir host. Therefore, the reservoir host responsible for the prevalence of the disease may still remain unknown.

With the end of the civil war for 30 years, people have started to move in to previously uninhabited areas. The recent increase of the prevalence of the disease may have been partly influenced by these population movements. In these parasites which may have maintained their transmission cycle with a help of a reservoir host will infect humans when they are exposed to these. If the dogs are the only reservoir host for the disease, the control of stray dogs, screening and treatment of domestic dogs may provide a significant contribution to the control of leishmaniasis. The real problem arises if the reservoir host is a forest living animal as the control of this animal is difficult. The increasing deforestation and establishment of human habitations in recent past may also have a significant impact on the increased prevalence of the disease as this may take humans closer to reservoir hosts. Further, increase in these activities may cause a significant health issue as in the case of Iran during 1999–2001 where an outbreak resulted due to increased construction buildings near rodent colonies [16].

## 7. Nature of the Parasite Species

Intracellular parasites of the genus* Leishmania* of family Trypanosomatidae are the parasites of this disease. So far, about 21 species of this genus have been identified as causative agents of the disease including* L. donovani* complex with 2 species,* L. donovani* and* L. infantum *(or* L. chagasi*);* L. mexicana* complex with 3 main species,* L. mexicana, L. amazonensis*, and* L. venezuelensis*;* L. tropica*;* L. major*;* L. braziliensis*;* L. guyanensis*;* L. panamensis*; and* L. peruviana* [5].* Leishmania donovani* complex is mainly responsible for the visceral form of the disease [19]. Out of these species* L. donovani* and* L. tropica* are the sources of anthroponotic infections, while most other species which cause cutaneous leishmaniasis act as sources of zoonotic infections. However, species of this complex also cause other types of the disease [5, 12].

In Sri Lanka* L. donovani* is the species that causes the cutaneous leishmaniasis [90]. The only strain isolated to date from skin lesions is* Leishmania donovani* MON-37, a zymodeme differing by only one nucleotide substitution in the glucose-6-phosphate dehydrogenase gene from* L. donovani* MON-2, the strain most commonly isolated from VL cases in India [26, 90]. Therefore, Sri Lanka is considered as the newest reported focus of leishmaniasis in the Indian subcontinent where the disease is caused by the most virulent visceralizing species,* L. donovani* [9]. Hence, the case has become more serious as a potential for visceralization in the cutaneous variant of* L. donovani* in Sri Lanka beginning to appear.

## 8. Lack of Proper Awareness about the Disease

Awareness about the disease in local community is one of the main factors that determines the success of a control program. It is essential to have the knowledge on symptoms for effective case management. In order to prevent infection by following preventive measures such as reducing the contact with potential reservoir animals, preventing the bite of sand flies by reducing the exposed parts of the body during outdoor works, use of insecticide impregnated mosquito nets, and managing household environment to reduce the sand fly populations, people must know about how the disease get transmitted (reservoir hosts and vectors), the peak biting time of the vector, and how to prevent the bite and reduce the sand fly populations. Surveys have shown that the presence of adequate knowledge has significant contribution to the success of control programs through changes in behavior and more widespread participation [91, 92].

However, according to a recent survey conducted in an endemic area for the disease in Anuradhapura District of Sri Lanka shows that the level of awareness on this disease among local population is very low. For an instance, 37% of the study population have had never heard of the disease even they were living in a endemic region for the disease and nearly half of the study population had no clue about any advanced diagnostic and treatment facilities [93]. On the other hand, around 20% of people believe that sand fly bite has a mild or no effect for their health according to this study. Hence, inadequate knowledge and awareness on the disease by communities living in the endemic areas is one of the challenges for control and prevention efforts.

## 9. Future Perspectives and Recommendations

Leishmaniasis can be considered as a significant health issue at global level. Therefore, institutions such as World Health Organization are now focusing on development of advanced diagnostic tests, drugs, and vaccines [69]. An increase of the use of advanced molecular technologies [87, 88] and studies on biological control methods such as use of microorganisms, frogs, mites, and spiders as biological control agents can be seen in the recent past [94, 95].

Immunization for the disease is not available since there is no proper vaccine available in the market for leishmaniasis. On the other hand, none of the existing treatment methods are ideal as each method has any complication such as high toxicity, resistance by the parasite, or low efficacy.

Lack of adequate scientific knowledge and literature based on the research work is one of the main problems for the prevention and control of the disease in Sri Lanka. However, considerable improvement of research interest about leishmaniasis can be seen in recent years. But, the problem comes when applying these scientific knowledge effectively in the control processes. First of all steps must be taken to increase the awareness among local communities. In this case it can be very effective if these awareness programs can be integrated to the general health care programs.

In South Asian countries, despite the fact that these are male dominant societies, mothers can be considered as the Centre of the family; therefore, the target personnel must be mothers of families. School students are the next most important target group as it is easier to educate them. Provision of knowledge and getting the community participation in control efforts will increase their success effectively.

On the other hand, extended researches on the identification of vectors and reservoir hosts, their biology, distribution, bionomics, and other important factors must be encouraged. Use of advanced techniques in researches may improve the effectiveness of these researches. For an instance, in the identification of reservoir hosts, screening each potential species is less effective and highly impractical; therefore, advanced and more reliable methods such as sand fly blood meal identification where blood meals of blood fed sand flies are screened for the presence of host DNA use molecular biological techniques [88]. As mentioned above growing information suggests that sand flies are not the only vectors for the disease but also species of sand flies of the genus* Sergentomyia* which was known to be harmless and are proven to be potential vectors. Sexual transmission is also possible for this disease.

Therefore, studies must be focused more and more on these subjects for proper identification of exact vectors and other means of transmission in order to take preventive and control measures. In both vector and reservoir control activities much higher attention must be given to environmental management and biological control strategies other than the use of chemicals. Studies on environmental requirements of vectors and reservoir animals will provide information needed for control of vectors and reservoirs by reducing preferable conditions through environmental management. Several biological control strategies have been suggested by studies in other countries. These methods can be adopted to suit our country after feasibility studies.

Geographical Information System (GIS) is an effective tool that can create distribution maps of diseases. These maps can be used to understand geographical distribution patterns of the disease and associated risk factors. Although, use of GIS mapping has been shown to be very effective in disease control by the studies in other countries [14, 96, 97], in Sri Lanka GIS is not much popular as a disease control tool. It is possible to ameliorate the efficiency in decision making and planning activities in the control of leishmaniasis by promoting these techniques more and more.

Leishmaniasis also has some serious complications such as post-kala-azar dermal leishmaniasis (PKDL) which is a complication of VL that results in nodular rashes in patients recovered from VL [73, 98] and diffuse cutaneous leishmaniasis (DCL), which is believed to be a result of an immunological defect of the human host [99]. Early diagnosis and continuous surveillance are very important to prevent such complications.

Psychological studies suggest that depression and anxiety are significantly higher in leishmaniasis patients [100]. Therefore, it is also important to pay attention to postdisease psychological effects, in the case of CL, especially, which does not cause death or a significant morbidity but leaves a lifelong scar in exposed parts of the body which affects the normal lifestyle of the patients even when they completely healed.

## 10. Conclusion

Unavailability of immunization due to the absence of a proper vaccine and the absence of a completely safe and effective treatment method indicates the importance of the control of the disease through prevention of the infection. Major challenge for this is the lack of knowledge about disease. Sufficient information especially on vectors, reservoir hosts, and vector behavior in terms of biting time, resting preferences, breeding, oviposition behavior, and host range is not adequately documented. In addition, only few studies have been conducted to find out the exact reservoir host for the* Leishmania* parasites in Sri Lanka, such as screening dogs and some other rodent species while there is a large range of mammals which may have the ability to act as probable reservoir hosts that exist in Sri Lanka. Complexity of the action of parasite is also a challengeable aspect. Lack of awareness among local communities is also another drawback for the success of the control of the disease. On the other hand, not having the access to advanced diagnostic techniques may affect negatively on the effectiveness of case management. However, studies must be carried out to reveal the biology, distribution, bionomics, and other important characters of vectors and reservoir hosts. Awareness programs must be conducted to improve the advocacy of the general public on the disease in order to enhance community participation in control activities while promoting the use of advanced technologies and techniques for diagnosis and continuous surveillance of the disease.

## Abbreviations

CL: Cutaneous leishmaniasis
MCL: Mucocutaneous leishmaniasis
VL: Visceral leishmaniasis
PCR: Polyclonal chain reaction
ELISA: Enzyme Linked Immunosorbent Assay
IFA: Indirect Fluorescent Antibody
DAT: Direct Agglutination Test
LST: Leishmanin Skin Test
PKDL: Post-kala-azar dermal leishmaniasis
DCL: Diffuse cutaneous leishmaniasis
IV: Intravenous
IM: Intramuscular
IL: Intralymphatic
GIS: Geographical Information System.
